# Supplementary material for: The Prognostic Effect of KRAS Mutations in Non-Small Cell Lung Carcinoma Revisited: A Norwegian Multicentre Study
Source: Cancers (Basel). 2021 Aug 26;13(17):4294. doi: 10.3390/cancers13174294 (PMC8428342; doi:10.3390/cancers13174294)
Supplement: Supplementary file 1 [file cancers-13-04294-s001.zip › cancers-1335599-supplementary.pdf]

cancers

MDPI

Table S1. Overview of first line curative and palliative treatments.

**Curative intention**	**Total, *n* = 671**		**I, *n* = 354**		**II, *n* = 147**		**III, *n* = 170**		**IV, *n* = 0**	
Complete resection + AT	163	(24.3)	27	(7.6)	58	(39.5)	78	(45.9)	-	-
Complete resection - AT	409	(61.0)	307	(86.7)	73	(49.7)	29	(17.1)	-	-
CT + RT <60 Gy	13	(1.9)	0	(0.0)	0	(0.0)	13	(7.6)	-	-
CT + RT >60 Gy	36	(5.4)	0	(0.0)	2	(1.4)	34	(20.0)	-	-
RT <60 Gy	10	(1.5)	3	(0.8)	3	(2.0)	4	(2.4)	-	-
RT >60 Gy	8	(1.2)	0	(0.0)	4	(2.7)	4	(2.4)	-	-
SBRT	24	(3.6)	17	(4.8)	5	(3.4)	2	(1.2)	-	-
Platinum doublet	8	(1.2)	0	(0.0)	2	(1.4)	6	(3.5)	-	-
**Palliative intention**	**Total, *n* = 400**		**I, *n* = 0**		**II, *n* = 0**		**III, *n* = 59**		**IV, *n* = 341**	
Complete resection + AT	9	(2.3)	-	-	-	-	0	(0.0)	9	(2.6)
Complete resection -AT	2	(0.5)	-	-	-	-	0	(0.0)	2	(0.6)
CT	137	(34.3)	-	-	-	-	21	(35.6)	116	(34.0)
RT	43	(10.8)	-	-	-	-	5	(8.5)	38	(11.1)
CT + RT	91	(22.8)	-	-	-	-	18	(30.5)	73	(21.4)
TKI	41	(10.3)	-	-	-	-	3	(5.1)	38	(11.1)
ICI monotherapy	40	(10.0)	-	-	-	-	8	(13.6)	32	(9.4)
ICI combination therapy	37	(9.3)	-	-	-	-	4	(6.8)	33	(9.7)
No treatment (% of total for each category)	46	(4.1)	5	(1.4)	1	(0.7)	1	(0.4)	39	(11.4)

Abbreviations: AT, adjuvant therapy; CT, chemotherapy; RT, radiotherapy; Gy, Gray; SBRT, sterotactic body radiation therapy; TKI, tyrosine inase inhibitor; ICI, immune checkpoint inhibitor.

Table S2. Associations between baseline patient characteristics and KRAS status, KRAS G12C, G12V and G12D.

Characteristic	KRAS wild type (*n* = 697)		KRAS mutated (*n* = 420)		*p*-value	G12C (*n* = 192)		G12V (*n* = 81)		G12D (*n* = 70)		*p*-value
Sex												
Female	353	(50.6)	239	(56.9)	0.042	114	(59.4)	39	(48.1)	44	(62.9)	0.136
Male	344	(49.4)	181	(43.1)		78	(40.6)	42	(51.9)	26	(37.1)	
Age (years												
<50	29	(4.2)	17	(4.0)	0.967	9	(4.7)	3	(3.7)	2	(2.9)	0.702
50–60	104	(14.9)	65	(15.5)		33	(17.2)	9	(11.1)	11	(15.7)	
>60	564	(80.9)	338	(80.5)		150	(78.1)	69	(85.2)	57	(81.4)	
Smoking history												
Never	113	(16.2)	13	(3.1)	<0.001	3	(1.6)	2	(2.5)	6	(8.6)	0.016
Former/current	584	(83.8)	407	(96.9)		189	(98.4)	79	(97.5)	64	(91.4)	
ECOG PS												
0–1	650	(93.3)	387	(92.1)	0.771	178	(92.7)	78	(96.3)	63	(90.0)	0.669
2	35	(5.0)	24	(5.7)		10	(5.2)	2	(2.5)	5	(7.1)	
3–4	12	(1.7)	9	(2.1)		4	(2.1)	1	(1.2)	2	(2.9)	
Disease stage												
I	218	(31.3)	141	(33.6)	0.763	69	(35.9)	25	(30.9)	23	(32.9)	0.790
II	94	(13.5)	54	(12.9)		26	(13.5)	12	(14.8)	7	(10.0)	
III	141	(20.2)	89	(21.2)		41	(21.4)	21	(25.9)	14	(20.0)	
IV	244	(35.0)	136	(32.4)		56	(29.2)	23	(28.4)	26	(37.1)	
Surgery												
No	339	(48.6)	195	(46.4)	0.474	83	(43.2)	33	(40.7)	32	(45.7)	0.827
Yes	358	(51.4)	225	(53.6)		109	(56.8)	48	(59.3)	38	(54.3)	
Number of metastatic sites at diagnosis												
No metastases	287	(41.3)	194	(46.4)	0.535	93	(48.7)	37	(45.7)	31	(44.9)	0.821
Local	199	(28.6)	104	(24.9)		48	(25.1)	25	(30.9)	17	(24.6)	
1 distant site	116	(16.7)	68	(16.3)		27	(14.1)	12	(14.8)	9	(13.0)	
2 distant sites	61	(8.8)	34	(8.1)		14	(7.3)	5	(6.2)	6	(8.7)	
3 or more distant sites	32	(4.6)	18	(4.3)		9	(4.7)	2	(2.5)	6	(8.7)	
No information	2	-	2	-		1	-	0	-	1	-	
Metastatic sites involved*												
Pleura												
No	603	(86.9)	391	(93.5)	<0.001	181	(94.3)	78	(96.3)	62	(91.2)	0.413
Yes	91	(13.1)	27	(6.5)		11	(5.7)	3	(3.7)	6	(8.8)	
No information	3	-	2	-		0	-	0	-	2	-	

Adrenal gland												
No	653	(94.0)	387	(92.4)	0.301	179	(93.2)	77	(95.1)	63	(91.3)	0.657
Yes	42	(6.0)	32	(7.6)		13	(6.8)	4	(4.9)	6	(8.7)	
No information	2	-	1	-		0	-	0	-	1	-	
Liver												
No	651	(93.7)	392	(93.6)	0.940	178	(92.7)	77	(95.1)	65	(94.2)	0.747
Yes	44	(6.3)	27	(6.4)		14	(7.3)	4	(4.9)	4	(5.8)	
No information	2	-	1	-		0	-	0	-	1	-	
Skeleton												
No	621	(89.4)	365	(87.1)	0.256	166	(86.5)	76	(93.8)	57	(82.6)	0.098
Yes	74	(10.6)	54	(12.9)		26	(13.5)	5	(6.2)	12	(17.4)	
No information	2	-	1	-		0	-	0	-	1	-	
Brain												
No	642	(92.4)	386	(92.1)	0.880	180	(93.8)	75	(92.6)	60	(87.0)	0.196
Yes	53	(7.6)	33	(7.9)		12	(6.3)	6	(7.4)	9	(13.0)	
	2	-	1	-		0	-	0	-	1	-	

Data are presented as *n* (%). Percentages are rounded and may not add up exactly to 100%. * cases with co-occurring metastases in other sites included. Abbreviations: PFS, progression free survival; OS, overall survival; HR, hazard ratio; ECOG PS, Eastern Cooperative Oncology Group performance status.

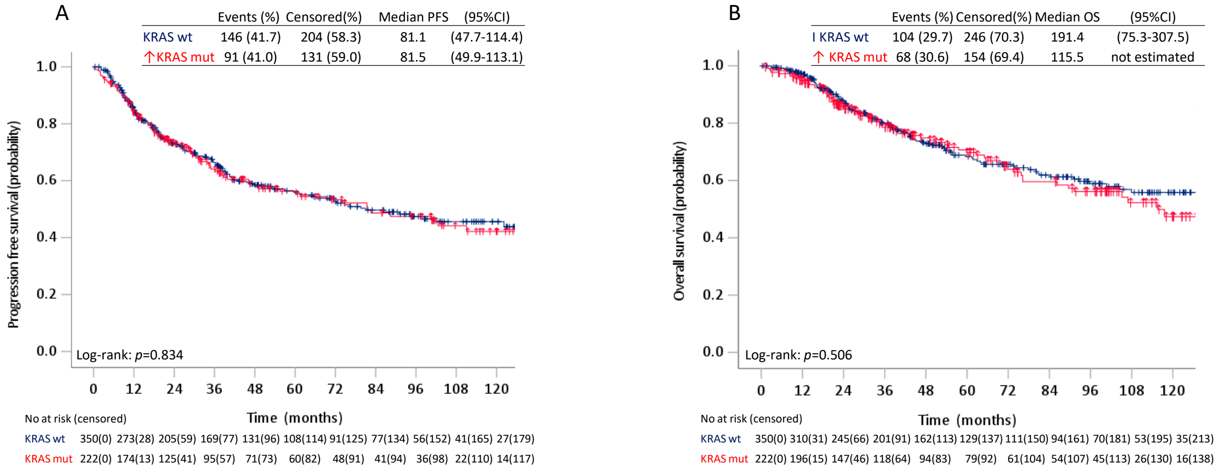

Figure S1. Progression free survival (A) and overall survival (B) in resected patients stage I-III with KRAS wild type and KRAS mutated tumours. .

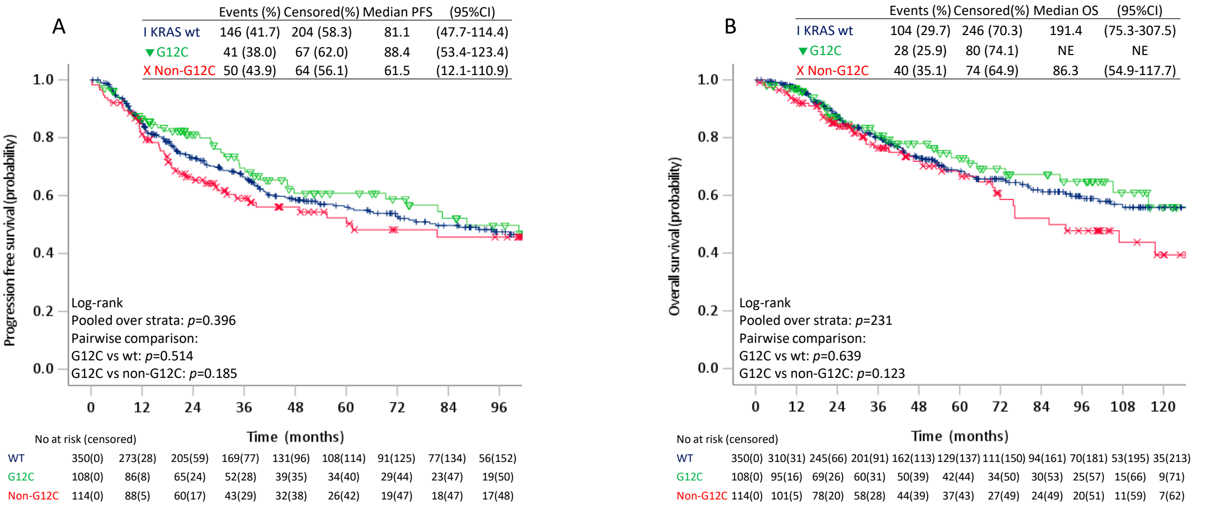

Figure S2. Progression free survival (A) and overall survival (B) in resected patients stage I-III with KRAS wild type, KRAS G12C and KRAS non-G12C mutated tumours.

Table S3. Multivariable analyses of progression free survival and overall survival in resected patients stage I-III with KRAS wild type, KRAS mutated (any mutation), KRAS G12C mutated.and KRAS non-G12C mutated tumours.

	PFS			OS			PFS			OS		
**Variable**	**HR**	**95%CI**	***p***	**HR**	**95%CI**	***p***	**HR**	**95%CI**	***p***	**HR**	**95%CI**	***p***
Sex												
Women	1(ref)			1(ref)			1(ref)			1(ref)		
Men	0.97	(0.75–1.26)	0.808	1.09	(0.80–1.48)	0.577	0.99	(0.76–1.28)	0.916	1.11	(0.82–1.51)	0.497
Age (years)	1.00	(0.99–1.02)	0.861	1.02	(1.01–1.05)	0.014	1.00	(0.98–1.02)	0.933	1.02	(1.00–1.04)	0.018
Smoking history												
Never	1(ref)			1(ref)			1(ref)			1(ref)		
Former/current	1.32	(0.85–2.03)	0.217	1.84	(1.05–3.23)	0.033	1.32	(0.86–2.04)	0.209	1.85	(1.05–3.24)	0.032
Disease stage												
I	1(ref)			1(ref)			1(ref)			1(ref)		
II	1.96	(1.40–2.74)	<0.001	1.67	(1.13–2.48)	0.010	1.98	(1.41–2.78)	<0.001	1.70	(1.15–2.52)	0.008
III	3.24	(2.22–4.73)	<0.001	2.60	(1.69–4.02)	<0.001	3.19	(2.18–4.66)	<0.001	2.55	(1.65–3.94)	<0.001
Adjuvant treatment												
No	1(ref)			1(ref)			1(ref)			1(ref)		
Yes	1.03	(0.73–1.46)	0.872	1.28	(0.86–1.91)	0.230	1.04	(0.73–1.47)	0.845	1.28	(0.86–1.92)	0.224
KRAS status												
Wild type	1(ref)			1(ref)			-	-	-	-	-	-
Mutated	0.97	(0.74–1.27)	0.844	1.03	(0.75–1.41)	0.866	-	-	-	-	-	-
KRAS G12C status												
Wild type	-	-	-	-	-	-	1(ref)			1(ref)		
G12C	-	-	-	-	-	-	0.85	(0.60–1.21)	0.359	0.86	(0.56–1.32)	0.495
KRAS non-G12C	-	-	-	-	-	-	1.11	(0.80–1.54)	0.540	1.19	(0.82–1.73)	0.370

Abbreviations: PFS, progression free survival; OS, overall survival; HR, hazard ratio; ECOG PS, Eastern Cooperative Oncology Group performance status.

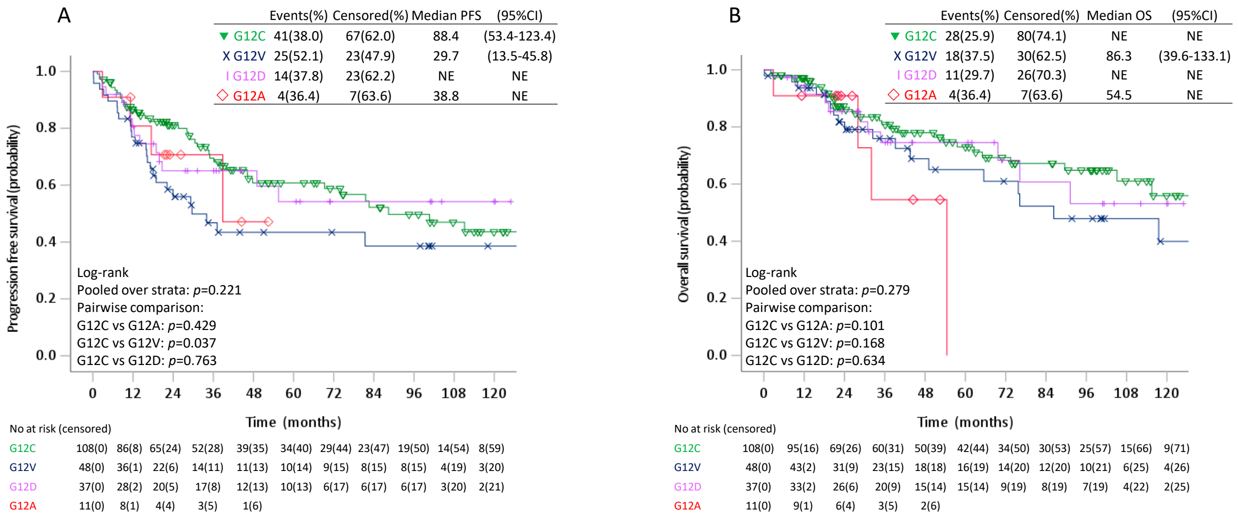

Figure S3. Progression free survival (A) and overall survival (B) in resected patients stage I-III with KRAS G12C, G12V, G12D and G12A mutated tumours.

Table S4. Multivariable analyses of PFS and OS in resected patients stage I-III with KRAS G12C and G12V mutated tumours.

Variable	HR	95%C)	*p*	HR	95%C)	*p*
Sex						
Female	1(ref)			1(ref)		
Male	0.85	(0.54–1.34)	0.475	0.89	(0.52–1.53)	0.677
Age (years)	1.00	(0.97–1.03)	0.811	1.00	(0.96–1.03)	0.768
Smoking history						
Never	1(ref)			1(ref)		
Former/current	1.72	(0.40–7.45)	0.467	2.28	(0.30–17.27)	0.426
Adjuvant treatment						
No	1(ref)			1(ref)		
Yes	1.04	(0.56–1.94)	0.898	0.83	(0.39–1.74)	0.613
Disease stage						
I	1(ref)			1(ref)		
II	1.38	(0.74–2.60)	0.316	1.18	(0.56–2.48)	0.657
III	2.75	(1.44–5.26)	0.002	2.06	(2.06–0.92)	0.078
KRAS mutation						
G12C	1(ref)			1(ref)		
G12V	1.43	(0.85–2.39)	0.180	1.32	(0.71–2.45)	0.378
G12D	1.16	(0.61–2.19)	0.655	1.23	(0.60–2.55)	0.576

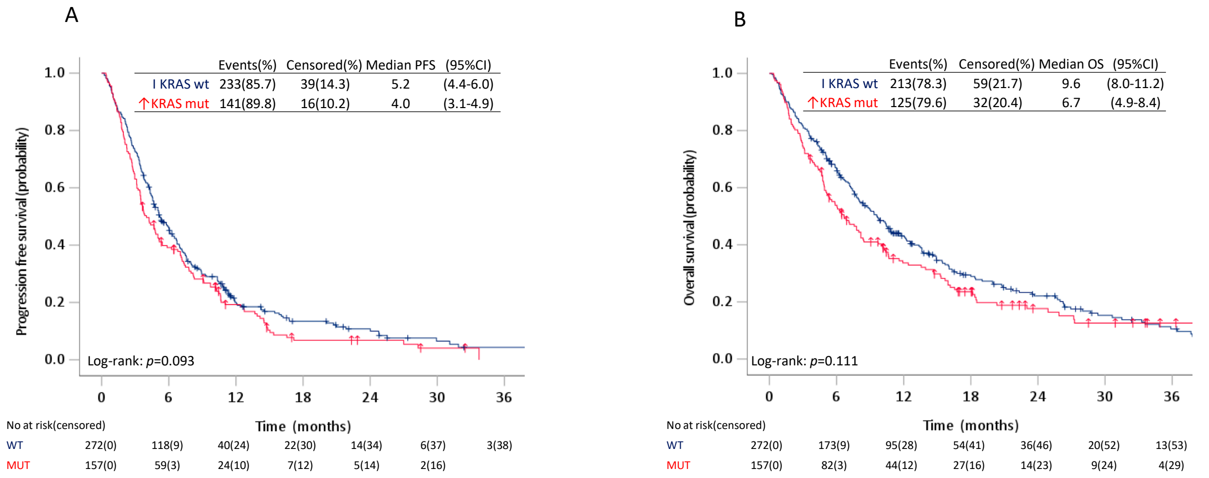

Figure S4. Progression free survival (A) and overall survival (B) in patients with advanced KRAS wild type and KRAS mutated tumours. .

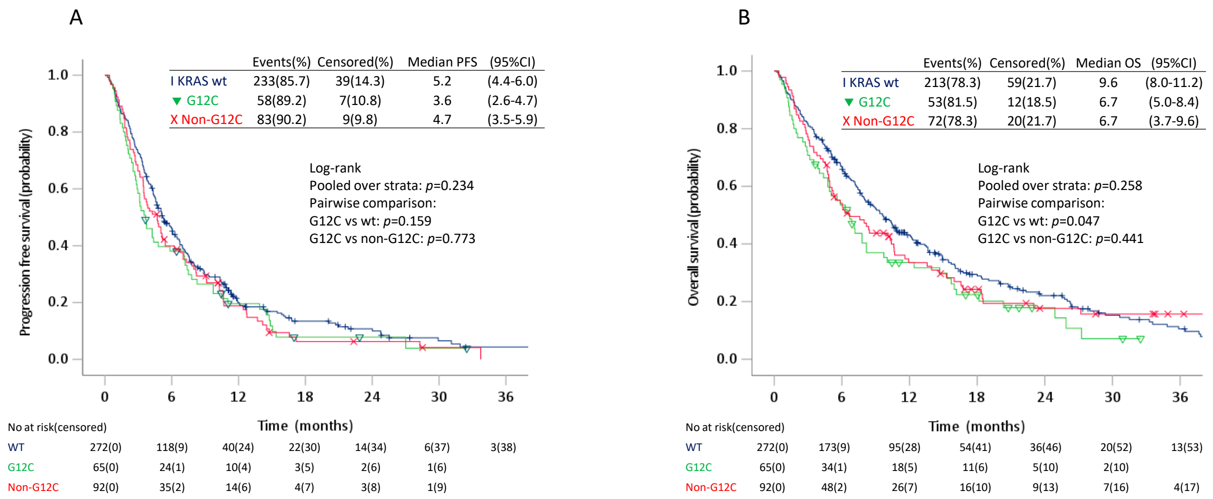

Figure S5. Progression free survival (A) and overall survival (B) in patients advanced KRAS wild type, KRAS G12C mutated and KRAS non-G12C mutated tumours.

Table S5. Multivariable analyses of progression free survival and overall survival in patients with advanced disease and KRAS wild type, KRAS mutated (any mutation), KRAS G12C mutated and KRAS non-G12C mutated tumours.

Variable	PFS			OS			PFS			OS		
	HR	95% CI	*p*	HR	95% CI	*p*	HR	95% CI	*p*	HR	95% CI	*p*
Age (years)	1.00	(0.99–1.01)	0.569	1.02	(1.00–1.03)	0.005	1.00	(0.99–1.01)	0.547	1.02	(1.00–1.03)	0.005
Sex												
Women	1(ref)			1(ref)			1(ref)			1(ref)		
Men	1.06	(0.86–1.32)	0.590	0.96	(0.77–1.20)	0.725	1.07	(0.86–1.33)	0.537	0.97	(0.77–1.21)	0.767
ECOG PS												
0–1	1(ref)			1(ref)			1(ref)			1(ref)		
2	1.58	(1.13–2.19)	0.007	2.12	(1.51–2.98)	<0.001	1.59	(1.15–2.22)	0.006	2.15	(1.53–3.04)	<0.001
3–4	4.33	(2.48–7.57)	<0.001	6.19	(3.45–11.10	<0.001	4.38	(2.50–7.65)	<0.001	6.18	(3.44–11.11)	<0.001
Smoking history												
Never smoker	1(ref)			1(ref)			1(ref)			1(ref)		
Former/current	1.44	(1.00–2.08)	0.052	1.38	(0.94–2.04)	0.103	1.43	(0.99–2.08)	0.055	1.38	(0.93–2.03)	0.107
Palliative CT first line												
No	1(ref)			1(ref)			1(ref)			1(ref)		
Yes	1.12	(0.84–1.50)	0.432	0.81	(0.69–1.10)	0.184	1.14	(0.85–1.52)	0.382	0.82	(0.60–1.12)	0.216
History of TKI (any line)												
No												
Yes	0.44	(0.30–0.64)	<0.001	0.35	(0.23–0.52)	<0.001	0.44	(0.30–0.65)	<0.001	0.35	(0.23–0.52)	<0.001
History of ICI (any line/combination)												
No	1(ref)			1(ref)			1(ref)			1(ref)		
Yes	0.60	(0.45–0.81)	0.001	0.45	(0.33–0.61)	<0.001	0.61	(0.45–0.81)	0.001	0.45	(0.33–0.62)	<0.001
Disease stage												
III	1(ref)			1(ref)			1(ref)			1(ref)		
IV	1.59	(1.17–2.17)	0.003	1.71	(1.23–2.39)	0.002	1.59	(1.17–2.16)	0.003	1.71	(1.22–2.39)	0.002
KRAS status												
Wild type							-	-	-	-	-	-
Mutated	0.97	(0.77–1.21)	0.784	1.05	(0.82–1.32)	0.715	-	-	-	-	-	-
KRAS G12C status												
Wild type	-	-	-	-	-	-	1(ref)			1(ref)		
G12C	-	-	-	-	-	-	1.06	(0.78–1.43)	0.726	1.16	(0.85–1.59)	0.346

KRAS non-G12C	-	-	-	-	-	-	0.92	(0.70–1.20)	0.522	0.97	(0.73–1.29)	0.842

Abbreviations: PFS progression free survival; OS overall survival; HR hazard ratio; ECOG PS Eastern Cooperative Oncology Group performance status; RT radiotherapy; CT chemotherapy; TKI tyrosine kinase inhibitor; ICI immune checkpoint inhibitor. .

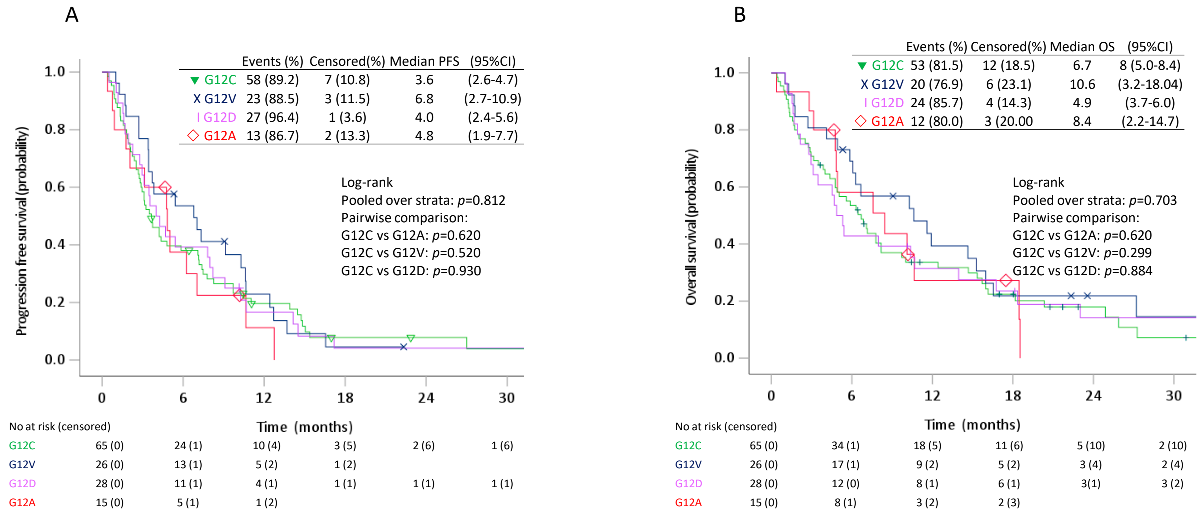

Figure S6. Progression free survival (A) and overall survival (B) in patients with advanced KRAS G12C, G12V, G12D and G12A mutated tumours.

Table S6. Multivariable analyses of progression free survival and overall survival in patients with advanced disease. and KRAS G12C, G12V and G12A mutated tumours.

Variable	PFS			OS		
	**HR**	**95% CI**	***p***	**HR**	**95% CI**	***p***
Age (years)	1.00	(0.98–1.02)	0.801	1.01	(0.99–1.04)	0.165
Sex						
Women	1 (ref)			1 (ref)		
Men	1.34	(0.89–2.00)	0.162	1.00	(0.65–1.54)	0.987
ECOG PS						
0–1	1 (ref)			1 (ref)		
2	1.50	(0.84–2.70)	0.171	1.91	(1.04–3.49)	0.036
3–4	3.22	(1.25–8.30)	0.015	5.51	(2.06–14.70)	0.001
Smoking history						
Never smoker	1 (ref)			1 (ref)		
Former/current smoker	0.56	(0.16–1.90)	0.349	0.51	(0.15–1.79)	0.295
Palliative CT and/or RT first line						
No	1 (ref)			1 (ref)		
Yes	0.99	(0.56–1.74)	0.964	0.87	(0.49–1.56)	0.650
History of TKI (any line) Yes/No						
No	1 (ref)			1 (ref)		
Yes	1.14	(0.46–2.83)	0.777	1.34	(0.52–3.42)	0.547
History of ICI (any line/ combination)						
No	1 (ref)			1 (ref)		
Yes	0.48	(0.28–0.84)	0.010	0.46	(0.27–0.80)	0.006
Disease stage						
III	1 (ref)			1 (ref)		
IV	1.03	(0.61–1.76)	0.900	1.19	(0.67–2.13)	0.558

KRAS mutation						
G12A	1 (ref)			1 (ref)		
G12C	0.74	(0.44–1.25)	0.261	0.70	(0.40–1.23)	0.215
G12V	0.87	(0.54–1.40)	0.563	0.90	(0.55–0.148)	0.681
G12D	1.14	(0.60–2.17)	0.695	0.90	(0.45–1.78)	0.755

Abbreviations: PFS, progression free survival; OS, overall survival; HR, hazard ratio; ECOG PS, Eastern Cooperative Oncology Group performance status; RT, radiotherapy; CT, chemotherapy; TKI, tyrosine kinase inhibitor; ICI, immune checkpoint inhibitor.
